# A retrospective study of long-term treatment outcomes for reduced vocal intensity in hypokinetic dysarthria

**DOI:** 10.1186/s12901-016-0022-8

**Published:** 2016-02-01

**Authors:** Christopher R. Watts

**Affiliations:** Davies School of Communication Sciences & Disorders, Texas Christian University, TCU Box 297450, Fort Worth, TX 76129 USA

**Keywords:** Voice, Voice disorders, Parkinson’s disease, Speech and language therapy

## Abstract

**Background:**

Reduced vocal intensity is a core impairment of hypokinetic dysarthria in Parkinson’s disease (PD). Speech treatments have been developed to rehabilitate the vocal subsystems underlying this impairment. Intensive treatment programs requiring high-intensity voice and speech exercises with clinician-guided prompting and feedback have been established as effective for improving vocal function. Less is known, however, regarding long-term outcomes of clinical benefit in speakers with PD who receive these treatments.

**Methods:**

A retrospective cohort design was utilized. Data from 78 patient files across a three year period were analyzed. All patients received a structured, intensive program of voice therapy focusing on speaking intent and loudness. The dependent variable for all analyses was vocal intensity in decibels (dBSPL). Vocal intensity during sustained vowel production, reading, and novel conversational speech was compared at pre-treatment, post-treatment, six month follow-up, and twelve month follow-up periods.

**Results:**

Statistically significant increases in vocal intensity were found at post-treatment, 6 months, and 12 month follow-up periods with intensity gains ranging from 5 to 17 dB depending on speaking condition and measurement period. Significant treatment effects were found in all three speaking conditions. Effect sizes for all outcome measures were large, suggesting a strong degree of practical significance.

**Conclusions:**

Significant increases in vocal intensity measured at 6 and 12 moth follow-up periods suggested that the sample of patients maintained treatment benefit for up to a year. These findings are supported by outcome studies reporting treatment outcomes within a few months post-treatment, in addition to prior studies that have reported long-term outcome results. The positive treatment outcomes experienced by the PD cohort in this study are consistent with treatment responses subsequent to other treatment approaches which focus on high-intensity, clinician guided motor learning for voice and speech production in PD. Theories regarding the underlying neurophysiological response to treatment will be discussed.

## Background

Among the physiological impairments resulting from Parkinson’s disease (PD) include the onset and progression of hypokinetic dysarthria. Hypokinetic dysarthria in PD is characterized by deviations in the rate, range, force, and tone of neuromuscular function in the muscles underlying speech production [1]. These deviations translate to effects on speech that include abnormalities in articulation and phonation. The classic clinical presentation of speech impairment in PD is characterized by a perceptually salient low volume and breathy voice quality, short rushes of speech, and imprecise articulation [2]. These vocal abnormalities result from impairments to neuromuscular control of respiratory and laryngeal muscles which numerous treatments, both medical and behavioral, have aimed to improve.

The pathophysiology of PD is linked to basal ganglia dysfunction and/or neural networks tied to this system. Unlike the limb effects of PD, however, evidence for pharmaceutical and surgical treatments improving hypokinetic dysarthria has been equivocal, suggesting that speech and voice manifestations of PD are influenced by pathways related to, but outside, the basal ganglia nuclei [3]. The model of PD progression proposed by Braak et al. (2004) suggested that early stage PD is characterized by neuronal impairment in the medulla and pons, including nuclei of the vagus and glossopharyngeal nerves [4]. Sapir (2014) has suggested that this model could explain why hypokinetic dysarthria is not sensitive to dopamine replacement therapy (cranial nerves are influenced by dopaminergic pathways, but do not directly utilize dopamine for neuronal communication) [3]. The muted effect of medication for treating the voice manifestations of PD, specifically the glottal incompetence resulting in low volume which progresses along with the disease, lends support to that position.

The laryngeal dysfunction resulting from hypokinetic dysarthria in PD is manifested by glottal incompetence due to bowing of the vocal folds, in some cases with accompanying atrophy [5]. The perceptual and physiological consequences of this impairment are reduced speech volume and vocal sound intensity, respectively. In theory these changes result from rigidity (Hypertonicity) in respiratory and laryngeal muscles due to the extrapyramidal dysfunction underlying the disease, although alternative theories of hypotonicity have also been presented [6–9]. In addition, alternative explanations for hypokinetic dysarthria tying voice and speech effects to factors other than rigidity have been proposed. Among these include impaired scaling of vocal effort resulting in the reduced vocal amplitude that is characteristic of speakers with PD [3]. This theory links the basal ganglia mediation of physical effort sense to the reduced vocal effort and subsequent low volume characterizing speech patterns of speakers with PD.

Bowed vocal folds are a characteristic of some speakers with PD, and surgical correction for the glottal incompetence has primarily involved injection laryngoplasty [10, 11]. Injectable substances are temporary, however, and repeated injections would be required for continuing improvement of glottal closure. Alternatively, a number of voice therapy approaches have demonstrated effective short and long-term outcomes for improving vocal amplitude and perceptual voice quality in populations with PD. A ubiquitous characteristic of voice therapy treatments for glottal incompetence, including those associated with PD, is a focus on high intensity (e.g., large number of repetitions) clinician guided exercise to promote adaptation in muscles and neurological pathways, and increased muscular effort to increase motor unit recruitment and the resulting amplitude of motor activity. These treatments target the issue of underscaling of vocal effort which is ubiquitous in speakers with PD [3].

A well-known evidence-based approach for treating the respiratory and laryngeal impairments in PD is the Lee-Silverman Voice Treatment (LSVT), now known as LSVT LOUD®. This structured intervention targets vocal effort scaling through increased vocal loudness via intensive, high effort vocalization and speech exercises designed to transfer to activities of daily living by improving neuromotor abilities and recalibrating the patients’ perception of effort during speech production [12]. LSVT utilizes a singular target and cueing strategy of “think loud” with the aim of facilitating neuromotor adaptation during speech production so that the elevated level of effort and resulting increased amplitude of motor activity becomes automatic and is perceived as natural by the patient. A number of modifications to the traditional LSVT method (16 treatment sessions over 4 consecutive weeks) have been described, including the employment of distance technologies and reduced frequency of sessions (e.g., 2× per week over 8 weeks), with similar reported treatment outcomes [13, 14]. Interestingly, the focus on increasing the amplitude of motor activity during LSVT has also been shown to improve articulation and swallowing abilities in some patients, reportedly due to carry-over effects in neuromotor abilities associated with structures and pathways tangentially trained in the LSVT exercises [15–17].

Vocal effort scaling and the underlying glottal incompetence in some speakers with PD has also been treated with other voice therapy approaches whose clinical goals relate to a similar focus on increased motor amplitude. A recent report described Phonation Resistance Training Exercise (PhoRTE) therapy applied to 60 individuals with glottal incompetence due to presbyphonia. The therapy tasks required of patients receiving PhoRTE were adapted from LSVT but differed in the frequency of treatments (1× per week instead of 4×), the incorporation of high pitch and low pitch productions of functional phrases, and a less rigorous home practice schedule. The authors reported significant improvements in participants’ perceptions of quality of life and perceived effort of voice production. These outcomes were similar to a comparison treatment, Vocal Function Exercises, and both experimental treatments resulted in greater clinical improvement compared to a control group who received no intervention [18].

Another related treatment focusing on vocal scaling, called “SPEAK OUT!®”, targets vocal effort by prompting patients to speak with “intent”, defined and modeled as a purposeful cognitive focus on increasing vocal loudness and intonation variability during speech [19]. Similar to LSVT, this treatment requires an intensive program although the number of treatment sessions is based on patient progress (e.g., 16 sessions are not required, as were in the original method for LSVT) and sessions last approximately 45 min. Each treatment session is structured with a hierarchy of speech, voice, and cognitive exercises progressing in the following manner: warm-up vocalizations → sustained vowel production → pitch glides → counting → reading → cognitive exercises. In the published literature, outcome data from only six patients receiving this treatment has been reported [20]. Reports which document clinical outcomes from larger samples, both in retrospective and prospective designs, will better inform clinical practice and evidence-based application of treatment approaches. The purpose of the present study was to investigate clinical outcomes in a large case series of patients who have received the SPEAK OUT! treatment in an effort to determine if measures of vocal function in patients with PD are positively or negatively impacted by this approach, and to compare results with the previously reported case reports.

## Methods

### Study design

This study used a retrospective design analyzing existing data from a consecutive case series of patients meeting inclusion criteria over a 3 year period. The primary outcome variable was vocal intensity (dBSPL) measured in three different speaking conditions: sustained vowel, reading, and conversation. Available data from records of patients within the cohort who were measured at 6 months and 1 year post-treatment was also collected. The methodology for this study was approved by a university Institutional Review Board (IRB# 1501–012–1501). The author has no competing interests to declare.

### Study population

Data for this study was collected from patient records of the clinical population at Parkinson Voice Project in Richardson, TX, who were treated between March 2011 and October 2014, who completed at least 12 treatment sessions and for whom pre-treatment and post-treatment data were recorded. All data came from patients diagnosed with idiopathic PD and who were experiencing vocal impairments. Of 100 consecutive patient files, 78 completed at least 12 treatment sessions before post-treatment measures were collected. Of the 22 who did not complete 12 treatments, reasons included (a) meeting treatment goals prior to 12 treatments or (b) illness or other life situations requiring withdrawal from treatment. When available, data was also recorded from post-treatment follow-up periods at approximately six months and twelve months.

### Description of the SPEAK OUT! therapy program

Data was recorded from patient files that underwent at least 12 treatment sessions. Each treatment was organized around a hierarchical framework through which a patient progressed during the course of a 45-minute (approximate) session and during home practice (once daily on treatment days, twice daily on non-treatment days). Clinicians administering treatment attended a training workshop specific to the intervention protocol which was administered by experienced clinicians, and each had more than two years of clinical experience. Each patient whose file was included in this study received three treatment sessions per week and completed homework exercises for which they returned homework logs at the subsequent treatment session. Each treatment and homework session followed the exact organizational framework, with stimuli printed in a therapy workbook provided to the patient who placed it open and in front of them during each session. The treatment hierarchy was as follows:

1. Warm-up vocalizations on nasal words (e.g., “may”, “me”, “my”)
2. Sustained vowel productions
3. Vowel pitch glides
4. Counting
5. Reading (phrases, sentences, and paragraphs)
6. Cognitive exercises (conversational speech) – these exercises provided written prompts to the patient in the form of carrier phrases which required the patient to complete in sentence form and then extend in conversation by providing the clinician with additional novel information about the topic. The cognitive exercises focused on improving word retrieval and processing speed. These responses required each patient to generate novel information while focusing on the treatment goal of speaking with intent.

The primary treatment goal and cueing strategy for treatment sessions was for each patient to speak with “intent”. Prior to the initiation of treatment, each participant was seen for a “Parkinson’s Information Session” in which the concept of “intent” was explained to them and treatment only began after a patient indicated understanding of the concept. “Intent” was defined as a purposeful cognitive focus in which the patient would direct attentional capacities on speech production. Cues such as “speak with authority,” “use your CEO voice,” and “say it with gusto” were associated with the concept of “intent” and utilized during treatment sessions. Confirmation of speaking intent included an increase in vocal loudness combined with variation of intonation which approximated more natural speech prosody during connected speech utterances. Patients were asked to speak with “intent” for every production throughout the treatment hierarchy. During treatment sessions, patients were cued by asking them to determine if prior utterances were produced with “intent” or not, and where appropriate “intent” was modeled for them by the clinician.

### Measures

Data for this study was collected from daily treatment logs of each patient, as recorded by the treating clinician. The Parkinson Voice Project has standardized their method of data collection during each treatment session, as follows:

- Patients were seated in front of a desk, behind which the clinician sat.
- A digital sound level meter (Radio Shack model 33–2055) was placed via a stand on the desktop in front of the patient, with the microphone head placed at arm’s length specific to each patient (determined by the patient extending their arm while seated comfortably, and the clinician placing the microphone head of the sound level meter at the patient’s wrist). This resulted in varied mouth-to-microphone distances between patients, but exact mouth-to-microphone distances within patients so that valid measures of vocal intensity could be compared across sessions. The interpatient variation in mouth-to-microphone distance was considered minimal, and the background noise in treatment rooms was less than 45dBSPL. The response rate of the sound level meter was set to fast.
- Patients were asked to produce any utterance while facing the clinician (with mouth directed toward microphone head of sound level meter).
- For each production within the hierarchical stages, the clinician recorded the minimum decibel level across the utterance duration (in dBSPL) on a daily record sheet.
- Dependent variables for this study included dB from three speaking conditions in the treatment hierarchy: sustained vowels, reading and conversation during the cognitive exercises. Mean minimum dB averaged across the respective utterance types at pre-treatment stages and post-treatment stages (e.g., data collected on the 12^th^ treatment session, at 6-months post-treatment follow-up and 12-months post-treatment follow-up) were analyzed.

### Statistical analysis

To compare treatment outcomes separate one-way multivariate analyses of variance (MANOVA) with repeated measures were applied to the pre-treatment and post-treatment data. Three separate MANOVA’s were used to compare pre-treatment to initial post-treatment measures, post-treatment at 6-months, and post-treatment at 12-months. In these statistical models, treatment time (pre vs. post) was the primary independent variable with decibel level in the three different speaking contexts (vowel, reading, conversation) as additional factors.

## Results

The mean age of the full sample was 71.3 years, which comprised 52 males (mean age = 72.9 years) and 26 females (mean age = 67.2 years). Mean years post-diagnosis onset at which treatment first began was 7.0 years. Table 1 presents group descriptive statistics across the dependent variables at the four measurement periods. Mean intensity increased across all three dependent variables after 12 treatment sessions by approximately 17dB, 9dB, and 6 dB for sustained vowels, reading, and conversation, respectively. These increases represented large effect sizes of *d* = 3.56 for sustained vowel, *d* = 3.09 for reading, and *d* = 2.58 for conversation.

**Table 1:** Descriptive statistics (mean and standard deviation in parentheses) from for the dependent variables (in dB) at the different measurement periods

Condition	Pre-Treatment	Post-Treatment	Follow-up 1	Follow-up 2
	*n* = 78	*n* = 78	*n* = 55	*n* = 30
Sustained Vowel	71.16 (6.01)	88.34 (3.23)	88.54 (4.91)	87.43 (6.39
Reading	67.57 (3.18)	76.54 (2.58)	73.26 (10.67)	75.00 (3.70)
Conversation	66.64 (2.85)	72.89 (1.90)	71.32 (2.33)	71.33 (2.62)

Pre- and post-treatment measures reflect data from 78 patients, Follow-up 1 (at 6 months) reflect data from 55 patients, and Follow-up 2 (at 12 months) reflect data from 30 participants

**Fig. 1:**
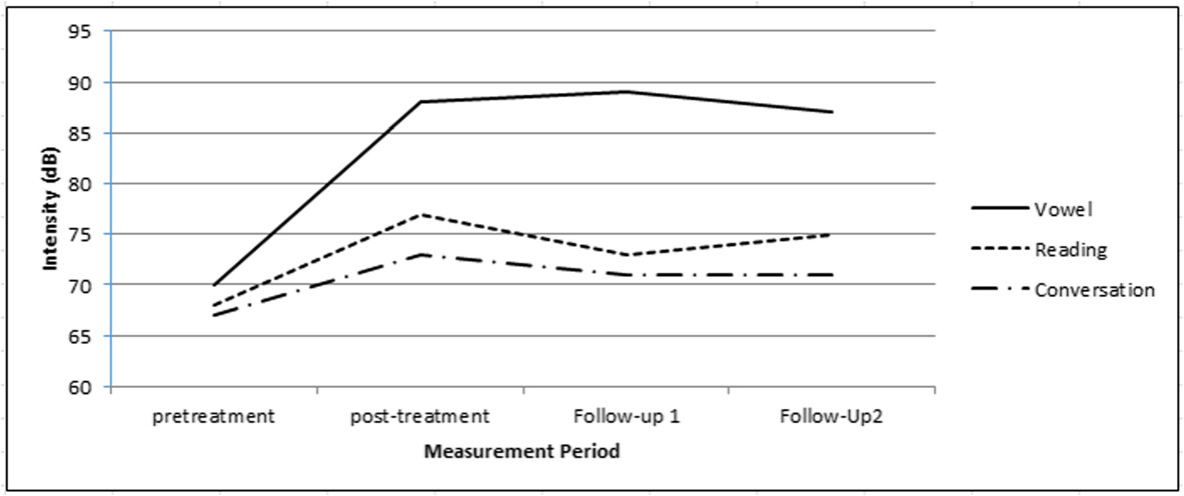
Mean minimum intensity (in dB) levels across the three speaking condition at four measurement timeframes: pre-treatment (*n* = 78), post-treatment (*n* = 78), Follow-up 1 @ 6-months (*n* = 55), and Follow-up 2 @ 12-months (*n* = 30)

From among this cohort 55 patients were measured at the 6-month follow-up period and 30 were measured again at the 12-month follow-up. Figure 1 illustrates mean intensity across the three speaking conditions at pre-treatment (*n* = 78), post-treatment (*n* = 78), the first follow-up (*n* = 55), and the second follow-up measurement periods (*n* = 30). At both follow-up periods speaking intensity remained above pre-treatment baseline levels. Effect sizes for mean intensity change comparing pre-treatment to the 6-month follow-up period remained large at *d* = 3.46, *d* = 0.75, and *d* = 1.87 for vowel, reading, and conversation, respectively. At the 12-month follow-up period effect sizes remained large at *d* = 2.90, *d* = 2.21, and *d* = 1.64 for vowel, reading, and conversation, respectively.

Three separate MANOVA’s were applied to the data to compare pre-treatment intensity levels to those at post-treatment, 6-month follow-up, and 12-month follow-up periods, respectively. For the pre-treatment vs. post-treatment analysis, there was a significant main effect for measurement period (Pillai’s Trace = 0.793, F[3,152] = 193.7, *p* < 0.001) with a corresponding large effect size as calculated by partial eta squared (η^2^ = 0.793), which reflects the error variance between the three speaking conditions as a percent variance explained. In this analysis there were significant treatment effects for sustained vowel (F[1,154] = 494.64, *p* < 0.001), reading (F[1,154] = 373.12, *p* < 0.001), and conversation (F[1,154] = 259.72, *p* < 0.001). For the pre-treatment vs. 6-month follow-up analysis there was a significant main effect for measurement period (Pillai’s Trace = 0.755, F[3,106] = 108.66, *p* < 0.001) with a corresponding large effect size (η^2^ = 0.755). In this analysis there were significant treatment effects for sustained vowel (F[1,108] = 329.06, *p* < 0.001), reading (F[1,108] = 15.27, *p* < 0.001), and conversation (F[1,108] = 95.72, *p* < 0.001). In the pre-treatment vs. 12-month follow-up analysis there was a significant main effect for measurement period (Pillai’s Trace = 0.692, F[3,56] = 42.03, *p* < 0.001) with a corresponding large effect size (η^2^ = 0.685). In this analysis there were significant treatment effects for sustained vowel (F[1,58] = 126.34,*p* < 0.001), reading (F[1,58] = 73.27, *p* < 0.001), and conversation (F[1,58] = 40.55, *p* < 0.001).

Collectively the statistical analyses revealed a significant treatment effect on vocal intensity measured at post-treatment, 6-month follow-up, and 12-month follow-up when compared to pre-treatment vocal intensity. In all comparisons vocal intensity increased as a result of treatment. In addition, the results revealed that intensity increased for all three speaking conditions (sustained vowel, reading, conversation) at post-treatment, 6-month follow-up, and 12-month follow-up, respectively, when compared to pre-treatment measurements. For all comparisons effect sizes were large, suggesting a strong degree of practical significance.

## Discussion

The purpose of this study was to investigate clinical outcomes in a large case series of patients who received an intensive program of speech therapy by measuring vocal intensity during sustained vowel, reading, and conversation at pre-treatment, post-treatment, and two follow-up periods. Findings revealed a significant treatment effect of on all measurements when compared to pre-treatment levels. These treatment effects were associated with large effect sizes. Collectively, the results of this study further support the notion that intensive speech and voice treatments focusing on vocal effort scaling are effective for increasing speaking intensity secondary to Parkinson’s disease. Additionally, results from this study suggested that treatment effects remained up to one-year post-treatment.

The largest treatment effect in this study was found on sustained vowel production. This was expected as both reading and conversation required connected speech with its variable intonation patterns resulting in a lower mean intensity across the utterances. The influence of speaking task on sound intensity and the differential influence of speaking task on response to treatment in patients with PD has also been demonstrated in prior studies [21]. Although long-term treatment effects across all speaking conditions are not unequivocal among previously reported investigations, the significant 6-month and 12-month follow-up effects found in this study are consistent with prior studies investigating long-term treatment effects secondary to LSVT [21, 22].

The significant post-treatment and long-term gains in vocal intensity subsequent to treatment are in line with outcomes from other evidence-based approaches which target reduced vocal intensity subsequent to glottal incompetence in PD or ageing. Interventions such as SPEAK OUT!, LSVT, and PhoRTE share methodological characteristics including high-intensity exercise protocols with clinician guided instruction and feedback to promote sustained motor learning. Theories explaining the neurophysiological changes subsequent to high-intensity vocal and speech exercises in PD have included improvement in glottal closure and/or vocal tension for increased sound pressure levels in speech, changes to extrapyramidal motor functions, and changes in limbic system pathways regulating goal-directed behavior [22]. A unique element of the treatment employed in the current study was the requirement for novel productions during conversational speech as a core element of the approach. The focus on “intent” is a method, similar to LSVT’s prompting of “think loud”, which helps the patient to rescale vocal effort during speech. In theory this may recruit and align cognitive pathways with the direct activation pyramidal pathways to facilitate increases in the number of motor units recruited in respiratory and laryngeal musculature during speech production. This hypothesis will need to be tested in future studies.

## Study limitations

This investigation was a retrospective study, which presents limitations on interpretation due to the nature of the research design. Important among these limitations were the inability to control for confounding factors that may have influenced within-subject responses to treatment (i.e., clinician differences, medication types/levels/schedule). Related to this, there was no comparison with a control group, so any improvement measured in this study may be the result of separate factors other than or in addition to the intervention. Due to the retrospective nature of the design treatment fidelity could not be assessed. Additionally, data from 22 patients among the initial cohort of 100 was not included in the final analysis due to lack of meeting full inclusion criteria, and subsequent intention-to-treat analysis was not performed. The patient cohort in this study also included males and females, although sex was not a factor in the study design. The degree to which sex influences outcomes will need to be addressed in subsequent experiments. This study did not include a rigid control over mouth-to-microphone distance, which is known to influence measurements of acoustic intensity. Prospective studies controlling for this factor are needed to further validate the results of this study. The outcome measures were not blinded to treatment or time point and were conducted by the treating clinicians, which could have led to measurement bias. The findings from this investigation will require validation from future prospective studies with designs controlling for the above mentioned limitations.

## Conclusion

This study investigated the effect of an intensive speech treatment focusing on rescaling of vocal effort to treat reduced vocal intensity due to hypokinetic dysarthria in PD. Retrospective data from 78 patients was analyzed to determine treatment effects after 12 therapy sessions (post-treatment), at a 6 month follow-up period, and at a 12 month follow-up period. Statistical analyses revealed significant treatment effects in the form of increased vocal intensity in sustained vowels, reading, and conversation at all three post-treatment measurement periods. These findings support the need for future prospective studies which control for additional factors as part of the scientific design. The positive treatment outcomes experienced by the PD cohort in this study are consistent with treatment responses subsequent to other treatment approaches which focus on high-intensity, clinician guided motor learning for voice and speech production in PD.

## Abbreviations

dBSPL: decibels in sound pressure level
IRB: Institutional Review Board
LSVT: Lee Silverman Voice Treatment
MANOVA: Multivariate Analysis of Variance
PD: Parkinson’s disease
PhoRTE: Phonation Resistance Training Exercise
